# Living Close to Natural Outdoor Environments in Four European Cities: Adults’ Contact with the Environments and Physical Activity

**DOI:** 10.3390/ijerph14101162

**Published:** 2017-09-30

**Authors:** Margarita Triguero-Mas, David Donaire-Gonzalez, Edmund Seto, Antònia Valentín, Graham Smith, David Martínez, Glòria Carrasco-Turigas, Daniel Masterson, Magdalena van den Berg, Albert Ambròs, Tania Martínez-Íñiguez, Audrius Dedele, Gemma Hurst, Naomi Ellis, Tomas Grazulevicius, Martin Voorsmit, Marta Cirach, Judith Cirac-Claveras, Wim Swart, Eddy Clasquin, Jolanda Maas, Wanda Wendel-Vos, Michael Jerrett, Regina Gražulevičienė, Hanneke Kruize, Christopher J. Gidlow, Mark J. Nieuwenhuijsen

**Affiliations:** 1 Centre for Research in Environmental Epidemiology (CREAL), ISGlobal, 08003 Barcelona, Spain; david.donaire@isglobal.org (D.D.-G.); antonia.valentin@isglobal.org (A.V.); david.martinez@isglobal.org (D.M.); gloria.carrasco@isglobal.org (G.C.-T.); albert.ambros@isglobal.org (A.A.); tania.martinez@isglobal.org (T.M.-I.); marta.cirach@isglobal.org (M.C.); txerms0@hotmail.com (J.C.-C.); mark.nieuwenhuijsen@isglobal.org (M.J.N.); 2 Universitat Pompeu Fabra (UPF), 08003 Barcelona, Spain; 3 CIBER Epidemiología y Salud Pública (CIBERESP), 08003 Barcelona, Spain; 4 Physical Activity and Sports Sciences Department, Fundació Blanquerna, Ramon Llull University, 08022 Barcelona, Spain; 5 Department of Environmental and Occupational Health Sciences, School of Public Health, University of Washington, Seattle, WA 98195, USA; eseto@uw.edu; 6 Centre for Sport, Health and Exercise Research, Staffordshire University, Stoke-on-Trent ST4 2 DE, UK, G.R.Smith@staffs.ac.uk (G.S.); daniel@happia.me (D.M.); G.L.Hurst@staffs.ac.uk (G.H.); n.j.ellis@staffs.ac.uk (N.E.); C.Gidlow@staffs.ac.uk (C.J.G.); 7 Department of Public and Occupational Health, Institute for Health and Care Research, VU University Medical Centre (VUMC), 1007 Amsterdam, The Netherlands; mm.vandenberg@vumc.nl (M.v.d.B.); martin.voorsmit@gmail.com (M.V.); e.clasquin@gmail.com (E.C.); jolandamaas@hotmail.com (J.M.); 8 Department of Environmental Science, Vytauto Didžiojo Universitetas, 44248 Kaunas, Lithuania; adedele@gmf.vdu.lt (A.D.); t.grazulevicius@gmail.com (T.G.); r.grazuleviciene@gmf.vdu.lt (R.G.); 9 Centre for Sustainability, Environment and Health, National Institute for Public Health and the Environment (RIVM), 3720 Bilthoven, The Netherlands; wim.swart@rivm.nl (W.S.); hanneke.kruize@rivm.nl (H.K.); 10 Centre for Nutrition, Prevention and Health Services, National Institute for Public Health and the Environment (RIVM), 3720 Bilthoven, The Netherlands; wanda.vos@rivm.nl; 11 Department of Environmental Health Sciences and Center for Occupational and Environmental Health, University of California, Los Angeles, CA 90095, USA; mjerrett@ucla.edu

**Keywords:** green spaces, physical activity, natural outdoor environments

## Abstract

This study investigated whether residential availability of natural outdoor environments (NOE) was associated with contact with NOE, overall physical activity and physical activity in NOE, in four different European cities using objective measures. A nested cross-sectional study was conducted in Barcelona (Spain); Stoke-on-Trent (United Kingdom); Doetinchem (The Netherlands); and Kaunas (Lithuania). Smartphones were used to collect information on the location and physical activity (overall and NOE) of around 100 residents of each city over seven days. We used Geographic Information Systems (GIS) to determine residential NOE availability (presence/absence of NOE within 300 m buffer from residence), contact with NOE (time spent in NOE), overall PA (total physical activity), NOE PA (total physical activity in NOE). Potential effect modifiers were investigated. Participants spent around 40 min in NOE and 80 min doing overall PA daily, of which 11% was in NOE. Having residential NOE availability was consistently linked with higher NOE contact during weekdays, but not to overall PA. Having residential NOE availability was related to NOE PA, especially for our Barcelona participants, people that lived in a city with low NOE availability.

## 1. Introduction

Interest in the health and physical activity (PA)-promoting potential of the physical environment continues to grow [1]. For example, some evidence suggests that the provision of natural outdoor environments (i.e., environments with vegetation like parks and environments with water like the seashore, abbreviated as NOE), the encouragement of certain types of food shops or the measures to reduce traffic density are ecological interventions that can affect health and activity behaviours [2,3]. 

Such higher level ecological interventions fall under the remit of those in charge of policy and the design and management of our living environments. Yet, there are no clear policy guidelines on the provision of NOE for health benefit that might inform the development of healthy cities. Residential NOE availability has been used as one indicator of whether a city is healthy or not [4,5,6], but based on limited evidence. Similarly, interest in the diverse urban planning needs of different population groups is increasing. For example, several studies suggest that women, lower-educated, children and elderly may use more NOE close to their residence than other population groups [7,8,9,10,11]. Despite no clear conclusions can be drawn from the existing evidence, usage differences could explain differential health benefits from NOE. Moreover, urbanity degree, ethnicity and location may introduce heterogeneity as well [9,12,13].

This study aimed to address these gaps using objective measures to understand possible associations between residential NOE availability, how much NOE are used, how much NOE are used for PA, and possible links with overall PA levels. We focused on objectively measured exposures and outcomes because we hypothesised that their findings would be more easily translated into policies than findings from subjective measures. Moreover, the use of objective or subjective measures is becoming a recognised cause of inconsistent results. Subjective and objective NOE availability assessments agree moderately [14]. However, the use of one or the other can lead to very different results (see [15] for an example). Similarly, as stated by Hagstromer et al. [16], subjective and objective measures of physical activity assess different things, with subjective measures usually assessing body movement and objective measures usually assessing effort. The correlations between subjective and objective measures of physical activity are usually low-to-moderate [16,17]. Usually, subjective measures are considered to be the best system to assess physical activity [16].

### 1.1. Contact with Natural Outdoor Environments

The links between access to and contact with (or use of) NOE is thought to be modified by factors like distance, weather, socio-cultural factors, and perceived safety [18]. Several studies have found that increasing residential NOE availability enhances contact [19,20,21,22,23,24]. However, to our knowledge, only one study investigated adults’ park visits using objective assessment of the NOE contact (specifically GPS-based measures) [25]. Evenson et al. [25] found that their participants spent around 95 min/week in NOE. Their sample was adults living, on average, around 600 m (0.4 miles) from a park, as the study focused only on this type of green spaces. So, to our knowledge, no study has evaluated if NOE contact is influenced by residential NOE proximity in adults using objective assessment of the NOE contact.

### 1.2. Overall Moderate-to-Vigorous Physical Activity

Regular PA prevents premature death and chronic diseases [26]. NOE could offer the opportunity for PA through providing locations that might be safe, accessible and attractive [2,3]. A review from 2008 found that parks and recreational settings availability increased PA in most studies, especially walking [27]. However, a 2011 review found that only 20 out of 50 studies reported a positive link between green spaces and PA, of which only three used objective PA indicators, all three focusing on children and using accelerometers to assess PA [28]. Similar no consistent findings were found in a review from 2015 focusing on objectively measured PA in the U.S. [29] that included studies using both accelerometers and pedometers to assess PA. From the 10 studies in adults, just two found positive relationships, another two found mixed results, and the other six found no associations. However, a recent study by Sallis et al. reported that the more parks near the respondents residence, the more physically active the respondents were [30]. Possible explanations for such inconsistent findings include use of different tools both to assess NOE availability [31,32,33,34,35,36,37,38,39] and PA [40,41,42], and diversity in study designs and settings [29,37]. 

### 1.3. NOE Moderate-to-Vigorous Physical Activity

One of the main gaps in the existing literature is that studies have rarely evaluated the actual use of NOE for PA, hypothesizing that PA was linked to NOE PA [25,37,43]. However, some findings indicate that NOE PA may be more beneficial to health than PA performed in other environments [44,45,46]. To our knowledge, only three studies have evaluated the association between residential availability of NOE and NOE PA [37,43,47] in adults, and none of them found relationships between NOE PA and NOE residential availability. In a sample of adults from four different European cities, we aimed to use objective measures to investigate whether residential NOE availability was linked to: contact with NOE, and moderate-to-vigorous PA (overall and in NOE separately). 

## 2. Materials and Methods

### 2.1. Study Sample

All participants were adults from a random sample of 3946 people aged 18–75 years in Europe as part of the Positive Health Effects on the Natural Outdoor Environment in Typical Populations of Different Regions in Europe (PHENOTYPE) project [13,48]. All 3946 people were invited to participate in this study. The only inclusion criterion was to be able to walk 300 m on level ground. In the case of Stoke-on-Trent, around half of the participants were from the original random sample and half were boosted through further mail sent to a random selection of households in the area and further opportunistic sampling within the area (for further details on data collection see Table A1). Participants were residents of four different cities: Barcelona (Spain, *n* = 107), Stoke-on-Trent (United Kingdom, *n* = 92), Doetinchem (The Netherlands, *n* = 105), and Kaunas (Lithuania, *n* = 104). Each participant provided written informed consent before taking part and received financial compensation on completion of the study. The study was conducted in accordance with the Declaration of Helsinki. Ethical approval was obtained from the corresponding authority in each city: Clinical Research Ethics Committee of the Municipal Health Care (CEIC PS-MAR), Spain (2012/4978/I); Staffordshire University Faculty of Health Science ethics committee, United Kingdom; Medical Ethical Committee of the University Medical Centre Utrecht, Netherlands; Lithuanian Bioethics Committee, Lithuania (2012-04-30 Nr.6B-12-147). 

### 2.2. Design

A detailed protocol was developed and followed in all participating cities. The protocol included instructions on smartphone placement and use. Accordingly, participants wore a smartphone with the CalFit application installed for seven consecutive days between May and December 2013. The smartphones were worn on a belt attached to the waist. Participants were instructed to remove the belt only when performing activities that could damage the smartphone (such as aquatic activities), when sleeping, and when it was necessary to charge the smartphone battery. The open-source CalFit software runs on Android operating system smartphones. CalFit uses the Global Positioning System (GPS) receivers in smartphones to collect valid information on location [49]. This information was used to determine the presence/absence of green spaces within a 50 m circular buffer of the participant location (Appendix A.1. CalFit data treatment). It has been recently reported that the median distance between coordinates acquired with Smartphone and with GPS trackers is 24 m overall [49]. Consequently, we used 50 m as a conservative approach to overcome this accuracy, so locations within 50 m from a NOE were considered to be in a NOE. CalFit uses the accelerometer motion sensor to assess PA intensity and duration and is in good agreement with the information collected with the widely used Actigraph accelerometer (concordance correlation coefficient, CCC, between 0.83 and 0.91) [49,50]. In the present study, CalFit was used to determine minutes of moderate-to-vigorous PA (MVPA, ≥3 METs) and time not wearing the smartphone. Episodes of 40 consecutive minutes or more with measures below 0.3 g in the vertical axis of CalFit were defined as non-wearing times. We investigated weekdays and weekends separately, hypothesising that relationships on days with everyday duties (i.e., working or studying) would be different than on days with available leisure time. For inclusion in analysis of weekdays, participants were required to have worn the smartphone for at least 10 h per day on three weekdays [50,51,52]. Similarly, for inclusion in analyses of weekends, 10 h per day on two weekend days were required. This resulted in a final sample of 350 participants on weekdays (86%) and 308 on weekend days (76%).

### 2.3. Measures

#### 2.3.1. Exposure

##### Residential Availability of Natural Outdoor Environments

Residential NOE availability was defined as the presence/absence of green spaces within 300 m of participants’ homes. The 300 m buffer was chosen for consistency with recommended indicators to be used across Europe [4,6], and on evidence that use of NOE might decline at distances greater than 300–400 m [53]. A 300 m network buffer was created around each participant residential address. To do so, we applied Network Analyst tools (ArcGIS10, Environmental Systems Research Institute (ESRI), Redlands, CA, USA) to the road network, excluding roads that were inaccessible to pedestrians. The presence/absence of green spaces within the buffers was derived from Urban Atlas 2006 [54] for three of the cities, and Top10 NL [55] for Doetinchem. Both used a 1:10,000 scale and minimum represented unit of 0.25 ha (Top10 NL was adapted to be consistent with Urban Atlas). The included NOE categories were urban green space, agricultural land, semi-natural areas, wetlands, and forests. 

#### 2.3.2. Outcomes

##### Contact with NOE

Contact with NOE was defined as daily average time spent in NOE, separately for weekdays and weekend days. This was derived from CalFit-recorded location data; for any given location point within the data recording period, participants were classified as being in NOE if there was a NOE within 50 m. If the point was inside the Urban Atlas city limits, we used Urban Atlas 2006 or Top10 NL (as above). For the all points that fell outside the city boundary, CORINE Land Cover 2006 (CLC2006) was used. 

##### Overall MVPA

Overall MVPA was based on CalFit-recorded accelerometer data. PA intensity was defined as the ratio of working metabolic rate to a standard resting metabolic rate (i.e., Metabolic Equivalent of Task, MET). We calculated the daily average time spent in MVPA, separately for weekdays and weekend days without MVPA duration restriction, following previous studies [30,56].

##### NOE MVPA

MVPA in NOE was derived from CalFit-recorded location points and time-matched accelerometer data. The indicator was calculated as the daily average time spent in MVPA in NOE based on the presence/absence of green or blue space within 50 m of each location point where PA was performed (as detailed under “Contact with NOE”). This was calculated separately for weekdays and weekend days without MVPA duration restriction, following previous studies [30,56].

#### 2.3.3. Covariates

We selected the following a priori covariates based on previous literature: gender [22,30,37,57,58], age [22,30,37,57,58], education completed [22,30,58], living with children younger than 11 years old [13], dog ownership [58], sampling season [58], and neighbourhood socioeconomic status (neighbourhood SES) [30,57]. Sampling season information was derived from sampling dates included in the analyses. All the rest of covariates were derived from information collected for a previous phase of the study [48]. As no comparable data between the four cities existed for neighbourhood SES, each city used its own local data [48].

### 2.4. Statistical Analyses

We conducted complete cases analyses separately for weekdays and weekend days without imputing missing data. Linear models were not considered appropriate after examining residual plots, so we fitted logistic regression models with adjustment for covariates to estimate the associations between residential NOE availability and each outcome separately. For our logistic regression models, we estimated goodness of fit with Hosmer-Lemeshow Test and measured predictive power with McFadden’s R2. We categorised our outcomes in two categories: value below or above median value of that variable in the city after excluding zeros. Categorised outcomes were: (i) low and (ii) high contact with NOE, (iii) low and (iv) high overall MVPA, (v) low and (vi) high NOE MVPA. Stratified analyses and interaction terms were included between residential NOE availability and (i) gender, (ii) age, and (iii) city to investigate effect modification. Statistical significance was set at *p*-value ≤ 0.05. R statistical package (version 3.1.0, R Foundation for Statistical Computing, Vienna, Austria) was used to carry out the analyses between 2015 and 2016.

### 2.5. Sensitivity Analyses

#### 2.5.1. Low Prevalence of Exposure Categories

Given the low prevalence of some of the exposure categories in Doetinchem (i.e., less than 4% of Doetinchem participants with absence of NOE at 300 m network buffer), we repeated the main analyses excluding this city to evaluate the robustness of our findings.

#### 2.5.2. Buffer Type for Abstracting NOE Indicators

To evaluate the robustness of findings to our selection of 300 m network buffer, we repeated the main analyses using exposure indicators for 150 m and 300 m Euclidean buffers and 500 m and 1000 m network buffers.

## 3. Results

### 3.1. Sample Characteristics

Participants differed from the original sample from which they were recruited; they were more physically active in all the cities and more highly educated in Stoke-on-Trent (data not shown). The characteristics of study participants, prevalence of outcomes, and description of indicators of natural outdoor environments are presented in Table 1. The participants of the different cities were statistically significantly different in most of the characteristics, with the exception of gender, living with children younger than 11 years, and neighbourhood socioeconomic status (Table 1, Table A2).

Our participants spent just over 40 min per day in NOE and around 80 min per day in overall MVPA (Table 1). NOE contact was statistically significantly higher for our Doetinchem participants than for the participants from the other cities (Table 1, Table A2). NOE contact was also higher on weekend days than on weekday in the pooled data and for Barcelona sample (Table A3). Conversely, overall MVPA was higher for our Barcelona participants than for the participants from the other cities during weekdays. During weekends, MVPA was statistically significantly lower for our Stoke-on-Trent sample than our Barcelona or Doetinchem samples (Table 1, Table A2). The pooled dataset and Barcelona and Stoke-on-Trent ones showed that participants spent statistically significantly more time doing MVPA during weekdays than on weekends (Table A3). 

Participants spent around eight minutes per day in NOE MVPA, with statistically significantly higher values in Doetinchem than in the other cities (Table 1, Table A2). Participants, therefore, performed around 9% of their MVPA in NOE. The only statistically significant differences found in the NOE MVPA between weekdays and weekend days, were in the pooled dataset (Table A3).

### 3.2. Contact with NOE

Having NOE in the 300 m buffer around the residence was statistically significantly associated with more NOE contact during weekdays, both in the pooled analyses and when stratified by age (Table 2 and Table 3). When stratified by gender or by city, the relationships only remained by females or for Barcelona participants (Table 4 and Table 5). The inclusion of an interaction between residential NOE availability and gender, or age, or city was not statistically significant (Table A4).

### 3.3. Overall MVPA

Residential NOE availability was not statistically significantly associated with overall MVPA duration in the pooled analyses (Table 2). This was unchanged when stratified by gender, by age and by city (Table 3, Table 4 and Table 5). However, on weekend days negative statistically significant links were found for our Kaunas sample (Table 4). The inclusion of the interaction between residential NOE availability and gender, age or city was not statistically significant (Table A4).

### 3.4. NOE MVPA

The higher residential NOE availability (i.e., having NOE in the 300 m buffer around the residence instead of not having it), the more NOE MVPA during weekdays. This was found both in the pooled analyses and when stratified by gender and by age (Table 2, Table 3 and Table 5). 

When stratifying by city (Table 4), having residential NOE availability was also statistically significantly associated with higher NOE MVPA for our Barcelona participants (both during weekdays and weekend days). Contrary, for our Kaunas sample, negative links were found during weekend days.

On weekdays, the inclusion of the interaction with city was statistically significant, while on weekend days interaction inclusion was statistically significant with age and city (Table A4).

### 3.5. Sensitivity Analyses

Sensitivity analyses showed consistent results (Table A5, Table A6, Table A7, Table A8 and Table A9). However, the effect of residential NOE availability on contact with NOE disappeared when investigating 1 km buffer sizes (Table A9). Similarly, the effect of having NOE around the residence on NOE MVPA vanished to marginally statistically significant in the models for 150 m buffer size (Table A7).

## 4. Discussion

We found that residential NOE availability was positively linked to NOE contact, when considering most of the week (i.e., weekdays). No associations were found between residential NOE availability and overall PA. Meanwhile, we found that the higher residential NOE availability, the more NOE PA, especially four our Barcelona participants.

### 4.1. Contact with NOE

Our data showed that having residential NOE availability was associated with higher NOE contact during most of the week (i.e., weekdays). This is in line with previous studies [19,21,22,23,57]. Our results show that this relationship is consistent using either objective or subjective measurement tools and across countries, as most of the previous studies have used questionnaires and have focused on northern countries. Our findings also indicate that people do not compensate a lack of NOE close to residence with fewer, longer visits to NOE that are further from the home [19].

### 4.2. Overall MVPA

We found no associations between residential NOE availability and overall MVPA. These results add to the current mixed evidence on the links between green spaces and objectively measured PA [29]. Sallis et al. [30] found that the higher number of parks at 500 m buffer around residence, the more MVPA in 14 cities from 10 different countries from around the globe. However, Sallis et al. adjusted their model for NOE availability and also by residential density, public transport density and pedestrian-accessible street intersections, which we were not able to do. Contrary, Carlson et al. [56] did not report links between number of parks and private recreation facilities within 500 m of residence and objectively measured MVPA (assessed with ActiGraph accelerometers) in different U.S. cities. In their study, Carlson et al. adjusted their models for walkability, aesthetics, walking facilities, social support, self-efficacy, barriers and several potential interactions between the previous factors. Interestingly, they found that MVPA was influenced by social support, self-efficacy and interactions between walkability and social support and between barriers and aesthetics. Similarly, no relationships were found between MVPA and green spaces indicators by Saelens et al. [59], but they found that higher MVPA was linked to commercial locations. 

Taking into account our finding and the previous evidence together, there is no suggestion for clear patterns for the links between residential NOE availability and overall PA between cities, population groups, or GIS-measured NOE indicators. Also, as noted by Bancroft et al. neighbourhood characteristics related with access to NOE (e.g., street configuration, accessibility, or crime) and NOE characteristics (e.g., aesthetics, safety, amenities, facilities, or perceived quality) could modify the association between overall PA and NOE, or even be better predictors of residents’ PA levels [29]. 

### 4.3. NOE MVPA

Our findings of residential NOE availability being tied to NOE MVPA during most of the week (i.e., weekdays) in pooled analyses and during the whole week (i.e., weekdays and weekends) for our Barcelona participants, contradict previous studies that did not find a link [37,43,60]. All the previous studies characterized natural environment availability using indicators of access to large NOE. Moreover, all of them were exploring the relationships in northern countries (i.e., countries between 50° and 60° of latitude). Thus their results are consistent with our lack of associations between access to large NOE in northern European cities (i.e., our Stoke-on-Trent, Kaunas and Doetinchem samples). Our results suggest that NOE PA is positively related with residential NOE availability, especially in those areas where NOE availability is low, as is the case of Barcelona, or the previously studied Odense in Denmark [43]. Ou et al. reported a positive link between PA and proximity to resident-preferred park, but no association with proximity to all parks or parks with sports/walking facilities [47]. We hypothesize that in areas with high NOE availability, greater choice results in more differences between proximity to resident-preferred NOE and proximity to nearest NOE. Meanwhile, in environments with low NOE availability, it is more likely that the nearest NOE will be the “preferred” park for residents. 

### 4.4. Strengths and Limitations

Our study is the first to use objective and standardized measures of NOE availability and objective measures of NOE contact and PA in four different European cities. This is also one of the first studies to evaluate objectively determined PA location. Consequently, this manuscript presents new findings that would be more easily translated into policies than findings from subjective measures.

Limitations of the study are as follows. First, causality cannot be inferred as our study has a nested cross-sectional design. Second, this was not a completely random sample. Participants were more physically active than the original sample from which they were recruited, so it seems that there was some self-selection bias. Consequently, our sample is not representative (especially not at city level). Third, there is the potential for measurement bias, as our PA measurement tool (CalFit) is less sensitive to certain activities that do not involve much vertical movement, such as cycling. This could be especially important for cities with a high percentage of cyclists like Doetinchem. Also, our NOE assessment (for residential availability, contact and NOE MVPA) was based on the mere presence, but we were not able to include real access (e.g., access points like doors) or quality indicators (e.g., safety). Moreover, the MVPA threshold we used (i.e., ≥3 METs) was not relative to population characteristics what, for example, could lead to the inclusion of light physical activity in our MVPA definition for those participants who were very fit. Fourth, our sample was not big enough to stratify by gender, age and city simultaneously, which restricted our capacity to identify their potential modifying effects. Finally, we were not able to collect enough information to study the role of the workplaces or commuting routes on people’s NOE contact and PA, despite some emerging evidence of their importance [61]. 

Future studies should involve different cities to provide a range of cultural contexts, with sufficiently large samples to allow stratification by gender, age, and city. Information and comparison on different NOE typologies (e.g., agricultural land compared with urban parks), quality of NOE, on NOE around workplaces and on contact with and MVPA performed close to residence would also be a valuable addition. 

## 5. Conclusions

Our study provides evidence that residential availability of natural outdoor environments is associated with more time spent in natural outdoor environments, but is not linked to overall duration of physical activity. Relationships between residential availability of natural outdoor environments and physical activity in natural outdoor environments were observed for our Barcelona sample, participants that live in a city with low availability of natural outdoor environments, but not for the other city samples. 

Policy makers should be cautious on using residential provision of natural outdoor environments to promote physical activity. Aside from physical activity, other health promoting aspects from the provision of natural outdoor environments should be explored.

## Figures and Tables

**Table 1:** Sample description and intercity comparisons using Kruskal-Wallis/Chi2 test.

	Total	Barcelona	Stoke-on-Trent	Doetinchem	Kaunas	Intercity Comparison
*Sample (n)*	408	107	92	105	104	
***Sociodemographic characteristics***						
*Gender*, females (*n* (%))	53.68%	46.73%	56.52%	57.14%	54.81%	
*Age* (years: median (IQR))	51.00 (26.00)	40.00 (23.00)	44.00 (29.00)	59.00 (16.00)	55.00 (23.50)	*
*Living with children <11 years old*, one or more (*n* (%))	19.90%	24.30%	25.27%	17.14%	13.46%	
*Dog ownership*, *yes* (*n* (%))	34.80%	23.36%	34.78%	22.86%	58.65%	*
*Highest education*, university or more (*n* (%))	56.76%	54.21%	47.25%	49.52%	75.00%	*
***Neighbourhood SES***						
Low	30.39%	40.19%	23.91%	30.48%	25.96%	
Medium	33.82%	35.51%	35.87%	29.52%	34.62%	
High	35.78%	24.30%	40.22%	40.00%	39.42%	
***Season*, autumn** (*n* (%))	51.12%	36.19%	54.35%	58.82%	55.77%	*
***Residential availability of natural outdoor environments***						
*Presence/absence of green spaces at 300 m network buffer*, one or more (*n* (%))	69.12%	41.12%	73.91%	96.19%	66.35%	*
***Weekdays***						
*Sample (n)*	350	101	70	93	86	
*Contact with NOE*, high (minutes: median (IQR))	41.40 (85.50)	14.67 (39.00)	32.23 (44.31)	114.60 (104.33)	40.30 (70.19)	*
*Overall moderate-to-vigorous physical activity*, high (minutes: median (IQR))	88.80 (57.58)	101.50 (59.50)	74.22 (68.28)	90.25 (53.50)	82.67 (42.89)	*
*NOE moderate-to-vigorous physical activity*, high (minutes: median (IQR))	7.73 (19.25)	4.20 (9.40)	4.60 (12.31)	21.00 (33.80)	8.57 (17.70)	*
***Weekends***						
*Sample (n)*	308	90	63	80	75	
*Contact with NOE*, high (minutes: median (IQR))	43.75 (122.50)	33.25 (94.50)	16.00 (33.50)	128.25 (119.00)	29.00 (102.00)	*
*Overall moderate-to-vigorous physical activity*, high (minutes: median (IQR))	78.25 (59.75)	88.75 (54.62)	53.00 (61.00)	81.50 (55.50)	74.50 (58.00)	*
*NOE moderate-to-vigorous physical activity*, high (minutes: median (IQR))	7.75 (24.12)	6.00 (15.88)	4.00 (10.50)	25.50 (31.75)	6.00 (19.25)	*

* Statistically significant differences (*p*-value ≤ 0.05) according to Chi2 or ANOVA tests. Notes: NOE for Natural Outdoor Environments. For contact with NOE, overall moderate-to-vigorous physical activity, and NOE moderate-to-vigorous physical activity (including both during weekday and weekends), the table is reporting the original data without categorisation.

**Table 2:** Adjusted models for residential NOE availability at 300 m network buffer.

Post-estimation measures/Model variables	Contact with NOE				*Overall Moderate-to-Vigorous Physical Activity*				*NOE Moderate-to-Vigorous Physical Activity*			
	Weekdays		Weekends		Weekdays		Weekends		Weekdays		Weekends	
	OR		OR		OR		OR		OR		OR	
	(95% CI)		(95% CI)		(95% CI)		(95% CI)		(95% CI)		(95% CI)	
**Post-estimation measures**												
R2 of the model	6%		2%		4%		1%		6%		3%	
Hosmer-Lemeshow test *p*-value	0.04		0.25		0.02		0.01		0.06		0.24	
**Model variables**												
Intercept	0.87 (0.32, 2.39)		1.29 (0.43, 3.88)		1.47 (0.54, 3.98)		2.85 (0.93, 8.69)		0.79 (0.28, 2.18)		1.40 (0.46, 4.22)	
Residential availability of NOE (one or more)	**2.41 (1.39, 4.17)**	*	0.93 (0.52, 1.67)		1.14 (0.67, 1.95)		0.82 (0.46, 1.45)		**2.42 (1.39, 4.22)**	*	1.12 (0.63, 2.02)	
City												
Stoke-on-Trent	0.78 (0.39, 1.56)		1.00 (0.48, 2.10)		0.96 (0.49, 1.91)		1.09 (0.53, 2.26)		0.90 (0.45, 1.82)		1.32 (0.63, 2.77)	
Doetinchem	0.95 (0.47, 1.95)		2.15 (1.00, 4.65)	*	1.23 (0.60, 2.53)		1.82 (0.85, 3.91)		0.93 (0.45, 1.92)		2.01 (0.92, 4.38)	
Kaunas	0.89 (0.45, 1.79)		1.32 (0.63, 2.74)		1.05 (0.53, 2.07)		1.12 (0.54, 2.31)		1.12 (0.56, 2.24)		1.36 (0.65, 2.86)	
Neighbourhood socioeconomic status												
Medium status	0.71 (0.41, 1.22)		0.84 (0.46, 1.53)		0.89 (0.52, 1.54)		1.09 (0.60, 1.98)		1.21 (0.69, 2.11)		0.94 (0.51, 1.71)	
High status	0.84 (0.48, 1.46)		1.28 (0.71, 2.33)		0.95 (0.55, 1.66)		1.03 (0.57, 1.88)		1.63 (0.93, 2.86)		1.02 (0.56, 1.85)	
Gender (female)	0.70 (0.45, 1.09)		0.66 (0.41, 1.07)		0.75 (0.48, 1.16)		0.58 (0.36, 0.93)	*	0.50 (0.32, 0.79)	*	0.74 (0.46, 1.19)	
Age	1.00 (0.98, 1.01)		0.98 (0.96, 1.00)	*	0.99 (0.98, 1.01)		0.97 (0.96, 0.99)	*	0.99 (0.98, 1.01)		0.98 (0.96, 1.00)	*
Education completed												
High level	1.33 (0.84, 2.11)		1.13 (0.69, 1.84)		0.89 (0.56, 1.39)		1.91 (1.17, 3.11)	*	0.86 (0.54, 1.37)		0.86 (0.53, 1.41)	
Sampling season (autumn)	0.76 (0.48, 1.20)		1.47 (0.90, 2.39)		0.68 (0.44, 1.08)		0.89 (0.55, 1.45)		0.83 (0.53, 1.32)		1.11 (0.68, 1.81)	
Dog ownership (yes)	1.35 (0.83, 2.20)		1.27 (0.75, 2.15)		1.82 (1.11, 2.96)	*	1.29 (0.77, 2.17)		1.42 (0.87, 2.31)		1.27 (0.75, 2.14)	
Living with children younger than 11 years old (yes)	0.88 (0.49, 1.56)		1.10 (0.60, 2.01)		1.97 (1.10, 3.53)	*	0.96 (0.52, 1.76)		0.73 (0.40, 1.31)		0.72 (0.39, 1.33)	

Notes: Models adjusted for neighbourhood socioeconomic status, city, gender, age, education level, sampling season, dog tenure and having children 11 years old or younger. Bold cells indicate those models where the relationship between the exposure and the outcome is statistically significant. Grey cells indicate those models where having residential NOE availability is statistically significantly associated to the outcome in the expected direction. NOE for Natural Outdoor Environments. * Statistically significant associations (*p*-value ≤ 0.05).

**Table 3:** Adjusted models for residential NOE availability at 300 m network buffer, stratified by age.

Outcomes	Below Median Age			Above Median Age		
	Estimate (95% CI)	*p*-Value		Estimate (95% CI)	*p*-Value	
***Contact with NOE***						
Weekdays	**2.28 (1.01, 5.12)**	**0.05**	^(1)^	**3.02 (1.32, 6.89)**	**0.01**	^(7)^
Weekend days	0.47 (0.20, 1.09)	0.08	^(2)^	2.00 (0.82, 4.91)	0.13	^(8)^
***Overall moderate-to-vigorous physical activity***						
Weekdays	1.18 (0.54, 2.59)	0.68	^(3)^	1.09 (0.51, 2.32)	0.83	^(9)^
Weekend days	0.71 (0.31, 1.62)	0.42	^(4)^	0.94 (0.41, 2.13)	0.88	^(10)^
***NOE moderate-to-vigorous physical activity***						
Weekdays	**3.05 (1.28, 7.27)**	**0.01**	^(5)^	**2.31 (1.07, 5.02)**	0.03	^(11)^
Weekend days	0.72 (0.31, 1.65)	0.44	^(6)^	2.45 (0.97, 6.19)	0.06	^(12)^

Notes: Models adjusted for neighbourhood socioeconomic status, city, gender, education level, sampling season, dog tenure and having children 11 years old or younger. For below median age: ^(1)^ R2 = 5%, Hosmer-Lemeshow *p*-value test = 0.27; ^(2)^ R2 = 3%, Hosmer-Lemeshow *p*-value test = 0.02; ^(3)^ R2 = 6%, Hosmer-Lemeshow *p*-value test < 0.01; ^(4)^ R2 = 3%, Hosmer-Lemeshow *p*-value test < 0.01; ^(5)^ R2 = 12%, Hosmer-Lemeshow *p*-value test = 0.60; ^(6)^ R2 = 3%, Hosmer-Lemeshow *p*-value test = 0.50. For above median age: ^(7)^ R2 = 12%, Hosmer-Lemeshow *p*-value test = 0.27; ^(8)^ R2 = 7%, Hosmer-Lemeshow *p*-value test = 0.73; ^(9)^ R2 = 4%, Hosmer-Lemeshow *p*-value test = 0.02; ^(10)^ R2 = −1%, Hosmer-Lemeshow *p*-value test < 0.01; ^(11)^ R2 = 6%, Hosmer-Lemeshow *p*-value test = 0.57; ^(12)^ R2 = 7%, Hosmer-Lemeshow *p*-value test = 0.55. Bold cells indicate those models where the association is statistically significant. Grey cells indicate those models where having residential NOE availability is statistically significantly associated to the outcome in the expected direction. NOE for Natural Outdoor Environments. Statistically significant associations (*p*-value ≤ 0.05).

**Table 4:** Adjusted models for residential NOE availability at 300 m network buffer, stratified by city.

Outcomes	Barcelona			Stoke-on-Trent			Doetinchem			Kaunas		
	OR	*p*-Value		OR	*p*-Value		OR	*p*-Value		OR	*p*-Value	
	(95% CI)			(95% CI)			(95% CI)			(95% CI)		
***Contact with NOE***												
Weekdays	**5.35 (2.05, 13.95)**	**<0.01**	^(1)^	1.34 (0.36, 4.92)	0.66	^(7)^	1.97 (0.15, 25.74)	0.61	^(13)^	0.77 (0.23, 2.63)	0.68	^(19)^
Weekend days	0.95 (0.35, 2.58)	0.92	^(2)^	2.05 (0.50, 8.39)	0.32	^(8)^	1.26 (0.09, 17.72)	0.87	^(14)^	0.39 (0.12, 1.29)	0.12	^(20)^
***Overall moderate-to-vigorous physical activity***												
Weekdays	1.23 (0.53, 2.90)	0.63	^(3)^	0.67 (0.18, 2.51)	0.55	^(9)^	1.05 (0.07, 15.19)	0.97	^(15)^	0.60 (0.20, 1.84)	0.37	^(21)^
Weekend days	0.85 (0.33, 2.17)	0.73	^(4)^	3.81 (0.88, 16.44)	0.07	^(10)^	0.52 (0.03, 7.97)	0.64	^(16)^	**0.18 (0.05, 0.66)**	**0.01**	^(22)^
***NOE moderate-to-vigorous physical activity***												
Weekdays	**7.62 (2.84, 20.40)**	**<0.01**	^(5)^	0.90 (0.20, 3.94)	0.89	^(11)^	1.17 (0.09, 15.73)	0.91	^(17)^	0.74 (0.23, 2.34)	0.61	^(23)^
Weekend days	**3.71 (1.23, 11.21)**	**0.02**	^(6)^	2.29 (0.54, 9.67)	0.26	^(12)^	1.36 (0.09, 19.35)	0.82	^(18)^	**0.19 (0.05, 0.68)**	**0.01**	^(24)^

Notes: Models adjusted for neighbourhood socioeconomic status, gender, age, education level, sampling season, dog tenure and having children 11 years old or younger. Pooled analyses also include city as a covariate. McFadden’s R2 range from <0.01 to 0.23. Hosmer-Lemeshow test results range from Chi2 = 39.59 (*p*-value < 0.01) to Chi2 = 1.60 (*p*-value = 0.99). For Barcelona: ^(1)^ R2 = 12%, Hosmer-Lemeshow *p*-value test = 0.06; ^(2)^ R2 = 7%, Hosmer-Lemeshow *p*-value test = 0.11; ^(3)^ R2 = 2%, Hosmer-Lemeshow *p*-value test = 0.15; ^(4)^: R2 < 1%, Hosmer-Lemeshow *p*-value test < 0.01; ^(5)^ R2 = 21%, Hosmer-Lemeshow *p*-value test = 0.31; ^(6)^ R2 = 19%, Hosmer-Lemeshow *p*-value test = 0.81. For Stoke-on-Trent: ^(7)^ R2 = 12%, Hosmer-Lemeshow *p*-value test = 0.57; ^(8)^ R2 = 7%, Hosmer-Lemeshow *p*-value test = 0.84; ^(9)^ R2 = 23%, Hosmer-Lemeshow *p*-value test < 0.01; ^(10)^ R2 = 17%, Hosmer-Lemeshow *p*-value test < 0.01; ^(11)^ R2 = 23%, Hosmer-Lemeshow *p*-value test = 0.75; ^(12)^ R2 = 7%, Hosmer-Lemeshow *p*-value test = 0.30. For Doetinchem: ^(13)^ R2 = 3%, Hosmer-Lemeshow *p*-value test = 0.35; ^(14)^ R2 = 5%, Hosmer-Lemeshow *p*-value test = 0.01; ^(15)^ R2 = 16%, Hosmer-Lemeshow *p*-value test < 0.01; ^(16)^ R2 = 11%, Hosmer-Lemeshow *p*-value test < 0.01; ^(17)^ R2 = 8%, Hosmer-Lemeshow *p*-value test < 0.01; ^(18)^ R2 = 4%, Hosmer-Lemeshow *p*-value test = 0.37. For Kaunas, ^(19)^ R2 = 19%, Hosmer-Lemeshow *p*-value test = 0.05; ^(20)^ R2 = 3%, Hosmer-Lemeshow *p*-value test = 0.66; ^(21)^ R2 = 7%, Hosmer-Lemeshow *p*-value test = 0.36; ^(22)^ R2 = 13%, Hosmer-Lemeshow *p*-value test < 0.01; ^(23)^ R2 = 9%, Hosmer-Lemeshow *p*-value test = 0.99; ^(24)^ R2 = 10%, Hosmer-Lemeshow *p*-value test = 0.93. Bold cells indicate those models where the association is statistically significant. Grey cells indicate those models where having residential NOE availability is statistically significantly associated to the outcome in the expected direction. NOE for Natural Outdoor Environments. Statistically significant associations (*p*-value ≤ 0.05).

**Table 5:** Adjusted models for residential NOE availability at 300 m network buffer, stratified by gender.

Outcomes	Males			Females		
	Estimate (95% CI)	*p*-Value		Estimate (95% CI)	*p*-Value	
***Contact with NOE***						
Weekdays	1.83 (0.84, 3.97)	0.13	^(1)^	**3.70 (1.55, 8.79)**	**<0.01**	^(7)^
Weekend days	0.69 (0.29, 1.63)	0.40	^(2)^	1.36 (0.58, 3.17)	0.48	^(8)^
***Overall moderate-to-vigorous physical activity***						
Weekdays	0.90 (0.41, 1.97)	0.79	^(3)^	1.59 (0.72, 3.49)	0.25	^(9)^
Weekend days	0.83 (0.35, 1.96)	0.68	^(4)^	0.71 (0.31, 1.63)	0.42	^(10)^
***NOE moderate-to-vigorous physical activity***						
Weekdays	**2.32 (1.06, 5.06)**	**0.04**	^(5)^	**2.63 (1.11, 6.24)**	**0.03**	^(11)^
Weekend days	2.10 (0.89, 4.98)	0.09	^(6)^	0.55 (0.22, 1.36)	0.19	^(12)^

Notes: Models adjusted for neighbourhood socioeconomic status, city, age, education level, sampling season, dog tenure and having children 11 years old or younger. For males: ^(1)^ R2 = 5%, Hosmer-Lemeshow *p*-value test < 0.01; ^(2)^ R2 = 5%, Hosmer-Lemeshow *p*-value test = 0.37; ^(3)^ R2 = 6%, Hosmer-Lemeshow *p*-value test < 0.01; ^(4)^ R2 = 8%, Hosmer-Lemeshow *p*-value test < 0.01; ^(5)^ R2 = 5%, Hosmer-Lemeshow *p*-value test = 0.61; ^(6)^ R2 = 5%, Hosmer-Lemeshow *p*-value test = 0.37. For females: ^(7)^ R2 = 6%, Hosmer-Lemeshow *p*-value test = 0.68; ^(8)^ R2 = 9%, Hosmer-Lemeshow *p*-value test = 0.57; ^(9)^ R2 = 4%, Hosmer-Lemeshow *p*-value test = 0.01; ^(10)^ R2 = 3%, Hosmer-Lemeshow *p*-value test = 0.02; ^(11)^ R2 = 11%, Hosmer-Lemeshow *p*-value test = 0.97; ^(12)^ R2 = 9%, Hosmer-Lemeshow *p*-value test = 0.57. Bold cells indicate those models where the association is statistically significant. Grey cells indicate those models where having residential NOE availability is statistically significantly associated to the outcome in the expected direction. NOE for Natural Outdoor Environments. Statistically significant associations (*p*-value ≤0.05)

## Appendix A

**Table A1:** Sampling strategy and participation details.

City	Invited *n*	Willing to Participate *n* (% from the Invited Ones)	Participated *n*	Finally Included in the Analyses
Barcelona	1044	379 (37%)	109	107
Stoke-on-Trent			99	92
From the original sample	1044	164 (17%)	49	45
Further approaches	4814	107 (2.22%)	50	47
Doetinchem	861	224 (26%)	111	105
Kaunas	997	280 (28%)	112	104

### Appendix A.1. CalFit Data Treatment

We downloaded CalFit data, including both accelerometer and location data from the smartphones and processed data in three steps.

(1) CalFit-recorded location data (including GPS and, when GPS data were not available, wireless network triangulation data) were converted into a Geographic Information Systems (GIS) data layer. We then attached the street network maps, and participant geocoded home and work addresses to the location data layer. After this, we resampled to 10 s to reduce the measurement error in the geolocation. Then we scanned the data sequentially looking for clusters of points based on the angular variability of its trajectory. This process identifies only the clearest clusters. Then, we made a spatial and temporal query around each point being part of a cluster to identify also the temporary closer points that are also spatially closer (i.e., points under the space and time threshold of 150 m and 30 min). The rest of points not being part of a cluster were considered trips. Finally we used the geocoded home and work points to identify the clusters belonging to these locations. The other clusters were considered others places and we calculated the centroid of each of them. The trips, the centroids and the geocoded home and work location points were the data used to continue with the analyses. The next step was to add information from Urban Atlas 2006 and Top10 NL and to develop the indicator of the presence/absence of green spaces within a 50 m circular buffer for each location point. Those location points identified as home or work were considered non-exposed to green spaces. Finally, we resampled to one-minute assigning the mode of all the calculated indicators. This resampling was done because one-minute was the minimum meaningful physical activity information that our measurement instruments could provide.
(2) CalFit-recorded accelerometer was used to get two g-forces (vertical and horizontal). After this, we converted the vertical force recorded in g-force into counts using a linear regression, and these counts into METs using the equation of Freedson et al. [62], as CalFit METs = 1.2907087 + (0.4141791 × VT g/min) [50]. We then defined time not wearing the CalFit as those periods of time of at least 40 consecutive minutes below 0.34 g in the vertical axis. These non-wear were excluded from analyses. We then classified those minutes with a MVPA intensity (≥3 METs).
(3) We excluded those days that were non-study days (e.g., delivery and collection days) and classified the remainder as weekdays or weekend days. We then applied the criteria of three days with at least 10 h as valid assessment for physical activity during weekdays and, similarly, applied two days with at least 10 h during weekend days [51,52]. Those participants not fulfilling the weekdays criteria were excluded from the weekdays analyses, while those participants not fulfilling the weekend days criteria were excluded from the weekend days analyses. This led to a total sample for this study of 350 participants on weekdays and 308 on weekend days (408 participants with either weekdays or weekend days data).

**Table A2:** Comparison of sample characteristics between the different cities. Results of Chi2, ANOVA, and posthoc Tukey, and Bonferroni tests.

Characteristics	Overall		BCN/SoT	BCN/Doe	BCN/Kau	SoT/Doe	SoT/Kau	Doe/Kau
	Chi2	*p*-Value	*p*-Value	*p*-Value	*p*-Value	*p*-Value	*p*-Value	*p*-Value
***Sociodemographic characteristics***								
*Gender*	2.94	0.40	-	-	-	-	-	-
*Age*	-	<0.01	0.05 ^‡^	<0.01 ^¥^	<0.01 ¤	<0.01 ^¥^	0.23	0.01 ^¥^
*Living with children < 11 years old*	6.15	0.10	-	-	-	-	-	-
*Dog ownership*	38.85	<0.01	0.51	1.00	<0.01 ¤	0.49	<0.01 ¤	<0.01 ¤
*Highest education*	19.97	<0.01	1.00	1.00	0.01 ¤	1.00	<0.01 ¤	<0.01 ¤
***Neighbourhood SES***	11.47	0.07	-	-	-	-	-	-
***Season***	13.07	<0.01	0.09	<0.01 ^¥^	0.03 ¤	1.00	1.00	1.00
***Residential availability of natural outdoor environments***								
*Presence/absence of green spaces at 300 m network buffer*	76.71	<0.01	<0.01 ^‡^	<0.01 ^¥^	<0.01 ¤	<0.01 ^¥^	1.00	<0.01 ^¥^
***Weekdays***								
*Contact with NOE*	-	<0.01	0.82	<0.01 ^¥^	0.06	<0.01 ^¥^	0.47	<0.01 ^¥^
*Overall moderate-to-vigorous physical activity*	-	<0.01	<0.01 ^†^	0.04 ^†^	<0.01 ^†^	0.71	1.00	0.80
*NOE moderate-to-vigorous physical activity*	-	<0.01	0.99	<0.01 ^¥^	0.50	<0.01 ^¥^	0.78	<0.01 ^¥^
***Weekends***								
*Contact with NOE*	-	<0.01	0.26	<0.01 ^¥^	0.83	<0.01 ^¥^	0.76	<0.01 ^¥^
			*Overall moderate-to-vigorous physical activity*	-	0.01	0.01 ^†^	0.97	0.29	0.04 ^¥^	0.50	0.57
*NOE moderate-to-vigorous physical activity*	-	<0.01	0.96	<0.01 ^¥^	1.00	<0.01 ^¥^	0.93	<0.01 ^¥^

Notes: Grey cells for those statistically significant tests. BCN for Barcelona, SoT for Stoke-on-Trent, Doe for Doetinchem and Kau for Kaunas. For contact with NOE, overall moderate-to-vigorous physical activity, and NOE moderate-to-vigorous physical activity (including both during weekday and weekends), the table is reporting the original data without categorisation. ^‡^ Indicate those variables with higher values in Stoke-on-Trent. ^†^ Indicate those variables with higher values in Barcelona. ^¥^ Indicate those variables with higher values in Doetinchem. ¤ Indicate those variables with higher values in Kaunas. NOE for Natural Outdoor Environments.

**Table A3:** Comparison of outcomes between weekdays and weekends. T-student tests results.

Outcomes	Pooled *p*-Value	Barcelona *p*-Value	Stoke-on-Trent *p*-Value	Doetinchem *p*-Value	Kaunas *p*-Value
***Contact with NOE***	**<0.01**	**<0.01**	0.12	0.91	0.23
***Overall moderate-to-vigorous physical activity***	*<0.01*	*<0.01*	*0.02*	0.48	0.14
***NOE moderate-to-vigorous physical activity***	**0.02**	0.06	0.48	0.20	0.55

Notes: For contact with NOE, overall moderate-to-vigorous physical activity, and NOE moderate-to-vigorous physical activity (including both during weekday and weekends), the table is reporting the original data without categorisation. Bold cells indicate that weekend values are higher than weekdays. Italics indicate that weekdays values are higher than weekends. NOE for Natural Outdoor Environments.

**Table A4:** Estimates of interaction terms (with 95% CI) and *p*-value of the likelihood ratio test comparing the model with and without the interaction term between residential NOE availability (defined as presence/absence of green spaces at 300 m network buffer) and gender, age, city.

Outcomes	Gender		Age		City			
	Female x residential NOE availability—Estimate (95% CI)	*p*-value of chi-2 test	Age above median age x residential NOE availability—Estimate (95% CI)	*p*-value of chi-2 test	Stoke-on-Trent x residential NOE availability—Estimate (95% CI)	Doetinchem x residential NOE availability	Kaunas x residential NOE availability—Estimate (95% CI)	*p*-value of chi-2 test
***Contact with NOE***								
Weekdays	0.81 (0.48, 1.38)	0.30	0.77 (0.44, 1.33)	0.51	0.51 (0.21, 1.22)	0.71 (0.32, 1.62)	0.58 (0.24, 1.40)	0.38
Weekend days	0.82 (0.46, 1.46)	0.20	0.74 (0.40, 1.34)	0.12	1.06 (0.42, 2.66)	2.03 (0.86, 4.83)	0.92 (0.36, 2.35)	0.28
***Overall moderate-to-vigorous physical activity***								
Weekdays	0.93 (0.55, 1.58)	0.15	0.69 (0.40, 1.19)	0.55	0.83 (0.35, 1.96)	1.12 (0.50, 2.52)	0.86 (0.36, 2.07)	0.89
Weekend days	0.56 (0.32, 1.01)	0.88	0.94 (0.52, 1.71)	0.36	1.46 (0.58, 3.68)	1.79 (0.75, 4.25)	0.82 (0.32, 2.07)	0.06
***NOE moderate-to-vigorous physical activity***								
Weekdays	0.57 (0.33, 0.97)	0.42	0.87 (0.50, 1.51)	0.64	0.45 (0.19, 1.10)	0.56 (0.25, 1.29)	0.52 (0.21, 1.26)	0.03
Weekend days	0.66 (0.37, 1.18)	0.52	1.10 (0.60, 1.99)	0.02	0.98 (0.40, 2.42)	1.37 (0.58, 3.21)	0.54 (0.21, 1.40)	0.01

**Table A5:** Sensitivity models. Adjusted models for residential NOE availability defined as presence/absence of green spaces at 300 m network buffer excluding Doetinchem.

Outcomes	Total	
	Estimate (95% CI)	*p*-Value
***Contact with NOE***		
Weekdays	**2.28 (1.29, 4.04)**	**<0.01**
Weekend days	0.88 (0.48, 1.62)	0.68
***Overall moderate-to-vigorous physical activity***		
Weekdays	1.06 (0.61, 1.84)	0.85
Weekend days	0.82 (0.45, 1.49)	0.52
***NOE moderate-to-vigorous physical activity***		
Weekdays	**2.42 (1.35, 4.32)**	**<0.01**
Weekend days	1.10 (0.59, 2.03)	0.77

Notes: Models adjusted for neighbourhood socioeconomic status, city, gender, age, education level, sampling season, dog tenure and having children 11 years old or younger. Bold cells indicate those models where the association is statistically significant. Grey cells indicate those models where having residential NOE availability is statistically significantly associated to the outcome in the expected direction. NOE for Natural Outdoor Environments. * Statistically significant associations (*p*-value ≤ 0.05)

**Table A6:** Sensitivity models. Adjusted models for residential NOE availability defined as presence/absence of green spaces at 300 m Euclidean buffer.

Outcomes	Total	
	Estimate (95% CI)	*p*-Value
***Contact with NOE***		
Weekdays	**2.62 (1.26, 5.44)**	**0.01**
Weekend days	1.30 (0.60, 2.83)	0.51
***Overall moderate-to-vigorous physical activity***		
Weekdays	1.35 (0.66, 2.76)	0.41
Weekend days	1.07 (0.50, 2.30)	0.86
***NOE moderate-to-vigorous physical activity***		
Weekdays	**4.19 (1.91, 9.18)**	**<0.01**
Weekend days	1.69 (0.75, 3.80)	0.20

Notes: Models adjusted for neighbourhood socioeconomic status, city, gender, age, education level, sampling season, dog tenure and having children 11 years old or younger. Bold cells indicate those models where the association is statistically significant. Grey cells indicate those models where having residential NOE availability is statistically significantly associated to the outcome in the expected direction. NOE for Natural Outdoor Environments. * Statistically significant associations (*p*-value ≤ 0.05).

**Table A7:** Sensitivity models. Adjusted models for residential NOE availability defined as presence/absence of green spaces at 150 m Euclidean buffer.

Outcomes	Total	
	Estimate (95% CI)	*p*-Value
***Contact with NOE***		
Weekdays	**1.82 (1.06, 3.12)**	**0.03**
Weekend days	1.61 (0.90, 2.88)	0.11
***Overall moderate-to-vigorous physical activity***		
Weekdays	0.69 (0.40, 1.18)	0.18
Weekend days	0.66 (0.37, 1.17)	0.15
***NOE moderate-to-vigorous physical activity***		
Weekdays	1.62 (0.94, 2.79)	0.08
Weekend days	1.37 (0.77, 2.45)	0.29

Notes: Models adjusted for neighbourhood socioeconomic status, city, gender, age, education level, sampling season, dog tenure and having children 11 years old or younger. Bold cells indicate those models where the association is statistically significant. Grey cells indicate those models where having residential NOE availability is statistically significantly associated to the outcome in the expected direction. NOE for Natural Outdoor Environments. * Statistically significant associations (*p*-value ≤ 0.05).

**Table A8:** Sensitivity models. Adjusted models for residential NOE availability defined as presence/absence of green spaces at 500 m network buffer.

Outcomes	Total	
	Estimate (95% CI)	*p*-Value
***Contact with NOE***		
Weekdays	**2.25 (1.14, 4.42)**	**0.02**
Weekend days	1.27 (0.62, 2.58)	0.52
***Overall moderate-to-vigorous physical activity***		
Weekdays	0.99 (0.51, 1.90)	0.97
Weekend days	0.78 (0.39, 1.57)	0.49
***NOE moderate-to-vigorous physical activity***		
Weekdays	**2.41 (1.21, 4.79)**	**0.01**
Weekend days	1.20 (0.58, 2.47)	0.62

Notes: Models adjusted for neighbourhood socioeconomic status, city, gender, age, education level, sampling season, dog tenure and having children 11 years old or younger. Bold cells indicate those models where the association is statistically significant. Grey cells indicate those models where having residential NOE availability is statistically significantly associated to the outcome in the expected direction. NOE for Natural Outdoor Environments. * Statistically significant associations (*p*-value ≤ 0.05).

**Table A9:** Sensitivity models. Adjusted models for residential NOE availability defined as presence of few/a lot of green spaces at 1000 m network buffer (with four green spaces as cut-off point).

Outcomes	Total	
	Estimate (95% CI)	*p*-Value
***Contact with NOE***		
Weekdays	1.39 (0.75, 2.59)	0.30
Weekend days	1.25 (0.61, 2.54)	0.54
***Overall moderate-to-vigorous physical activity***		
Weekdays	0.75 (0.40, 1.39)	0.36
Weekend days	1.00 (0.50, 2.00)	0.99
***NOE moderate-to-vigorous physical activity***		
Weekdays	**1.94 (1.02, 3.70)**	**0.04**
Weekend days	1.49 (0.71, 3.13)	0.29

Notes: Models adjusted for neighbourhood socioeconomic status, city, gender, age, education level, sampling season, dog tenure and having children 11 years old or younger. Bold cells indicate those models where the association is statistically significant. Grey cells indicate those models where having residential NOE availability is statistically significantly associated to the outcome in the expected direction. NOE for Natural Outdoor Environments. * Statistically significant associations (*p*-value ≤0.05).
